# New pecJ-*n* (*n* = 1, 2) Basis Sets for Selenium Atom Purposed for the Calculations of NMR Spin–Spin Coupling Constants Involving Selenium

**DOI:** 10.3390/ijms24097841

**Published:** 2023-04-25

**Authors:** Yuriy Yu. Rusakov, Irina L. Rusakova

**Affiliations:** A. E. Favorsky Irkutsk Institute of Chemistry, Siberian Branch of the Russian Academy of Sciences, Favorsky St. 1, 664033 Irkutsk, Russia; rusakov82@mail.ru

**Keywords:** J-oriented basis set, NMR, spin–spin coupling constant, selenium, pecJ-1, pecJ-2

## Abstract

We present new compact pecJ-*n* (*n* = 1, 2) basis sets for the selenium atom developed for the quantum–chemical calculations of NMR spin–spin coupling constants (SSCCs) involving selenium nuclei. These basis sets were obtained at the second order polarization propagator approximation with coupled cluster singles and doubles amplitudes (SOPPA(CCSD)) level with the property-energy consistent (PEC) method, which was introduced in our previous papers. The existing SSCC-oriented selenium basis sets are rather large in size, while the PEC method gives more compact basis sets that are capable of providing accuracy comparable to that reached using the property-oriented basis sets of larger sizes generated with a standard even-tempered technique. This is due to the fact that the PEC method is very different in its essence from the even-tempered approaches. It generates new exponents through the total optimization of angular spaces of trial basis sets with respect to the property under consideration and the total molecular energy. New basis sets were tested on the coupled cluster singles and doubles (CCSD) calculations of SSCCs involving selenium in the representative series of molecules, taking into account relativistic, solvent, and vibrational corrections. The comparison with the experiment showed that the accuracy of the results obtained with the pecJ-2 basis set is almost the same as that provided by a significantly larger basis set, aug-cc-pVTZ-J, while that achieved with a very compact pecJ-1 basis set is only slightly inferior to the accuracy provided by the former.

## 1. Introduction

Selenium chemistry plays an important role in modern materials science and has many applications in modern industries, from photosensitive electronics to pharmaceuticals. For instance, selenium is known to be an essential element in biochemistry [1,2,3,4,5,6,7,8,9]; in particular, it is embedded in selenoproteins as the 21st amino acid selenocysteine [10]. Selenium compounds have also found their applications in photoelectronics [11,12], solar cells technology [13], the semiconductors industry [14], nanotechnology [15,16,17], the synthesis of channelized porous materials [18,19], and in many other fields. Today, any synthesis of novel selenium-containing compounds involves NMR experiments supplemented by high-quality quantum chemical modeling of their NMR spectra [20,21,22,23,24,25,26,27,28]. In this respect, a delicate approach is required for the calculation of the indirect nuclear spin–spin coupling constants (SSCCs) involving selenium [29,30,31,32] because the calculation of the SSCCs with heavy chalcogens from the fourth period and below, in general, represents a challenging task [33,34,35,36,37,38,39,40,41] due to the complexity of the electronic structure of such systems and the need to pay special attention to the relativistic effects [42,43,44,45]. Accurate computation of the selenium SSCCs covers a wide range of applicability, representing, in the first place, a powerful tool of the stereochemical studies of organoselenium compounds [46,47,48,49,50,51,52,53,54]. Indeed, it should be emphasized that Se-H and Se-C SSCCs manifest a pronounced stereochemical behavior basically governed by the orientational lone-pair effect of selenium [48,55]. For example, large and, in some cases, even dramatic differences between geminal and vicinal Se-H SSCCs involving diastereotopic protons allowed making unambiguous diastereotopic assignments of signals in the ^77^Se-^1^H HMBC spectra of the series of selenium-containing four-, five-, and six-membered heterocycles, including the derivatives of thiaselenetane, selenasilole, thiaselenole, thiaselenolane, and dihydrothiaselenine [54]. Another example of the usefulness of the accurately computed ^n^*J*(^77^Se,^1^H) SSCCs was presented in the work [49], where these were used for the correct assignment of the signals in the ^1^H-^77^Se HMBC spectra of Se-glycosides and related molecules, allowing the determination of their glycosidic conformation around the C–Se bond. Stereochemical studies of nine *Z*-2-(vinylsulfanyl)ethenylselanyl organyl sulfides have been carried out by means of NMR experimental measurements and high-quality computations of the ^77^Se-^1^H spin–spin coupling constants [50]. Unambiguous resonance assignments of diastereotopic CH_2_ protons in the anomeric side chain of nine alkyl- and aralkylselenoglycosides have been carried out on the basis of experimental CPMG-HSQMBC measurements and high-quality computations of geminal ^77^Se-^1^H spin–spin coupling constants involving diastereotopic pro-R and pro-S protons [51]. Such examples are numerous, inferring the importance of the accurate prediction of the SSCCs involving selenium using high-quality quantum chemical methods and properly chosen basis sets.

In this respect, many accurate quantum–chemical approaches to the calculations of SSCCs have been developed over the past few decades [56,57,58,59,60,61,62]. The most popular ones comprise the multi-configurational self-consistent field approach (MCSCF) [63,64], the density functional theory (DFT) [65,66,67,68,69], the coupled cluster approach (CC) [70,71,72,73,74,75], and the second-order polarization propagator approximation (SOPPA) [76,77] along with its modifications, the SOPPA(CC2) [78] and SOPPA(CCSD) [79,80], obtained using the single and double amplitudes from the coupled-cluster calculations with the CC2 [74] and CCSD [70,75] models, respectively. 

Significantly, even if a high-quality ab initio approach has been used when calculating the SSCCs, it does not guarantee the correctness of the result. There are many factors affecting the final values, though the most important one is the quality of the basis set used. Regardless of what high-quality approach was used, a small and inflexible basis set will poorly describe the main contributions to the SSCCs and will spoil the whole calculous. In particular, it is well known that standard energy-optimized basis sets are not effective for the calculation of the second- and higher-order molecular properties, such as NMR SSCCs. This is because such basis sets do not usually provide an appropriate representation of the molecular orbitals in the desired areas and provide slow convergence towards the complete basis set (CBS) limit. As a consequence, to achieve values close to the CBS limit within a particular method, one is forced to resort to rather large nonspecialized basis sets, resulting in high computational demands. This problem is especially pronounced for the SSCCs involving nuclei of fourth and further periods of the periodic table of elements (PTE) because of the complexity of their atomic shells requiring a more flexible description. In this way, specialized *J*-oriented basis sets are under permanent development. Such basis sets usually reflect the peculiarities of the SSCCs, which follow from the simplest nonrelativistic Ramsey formulation [81]. Each of the four Ramsey contributions to SSCCs, namely, Fermi contact (FC), the spin dipolar (SD), the paramagnetic spin-orbit (PSO), and the diamagnetic spin-orbit (DSO), require basis set flexibility in different exponential regions [82,83]. The most proliferated case is when the FC term predominates over the other three contributions; thus, a majority of *J*-oriented basis sets were developed so as to improve the description of the FC term only. In that case, the ultimate goal of such basis sets was to enhance the description of the *s*-electron density at the nuclear positions by extending the standard energy-optimized basis sets with tight *s*-type functions with very high exponents [80]. This approach can be thought of as the most straightforward one because it is based on the consecutive augmentation of the important angular spaces using a recurrent ratio (geometrical progression, at most). In this respect, it should be mentioned that such an approach was used to obtain the now-popular aug-cc-pVTZ-J series of basis sets for different elements [80,84,85,86,87,88,89,90,91]; the Pople-style basis sets 6-31G-J and 6-311G-J [92]; the acvXz-J for Se, Te, and Sn [93,94]; and the av3z-J for Te [41]. The other approaches involve, in some way or another, the optimization procedure, namely the search for the values of additional exponents is carried out via the variational procedure. In this way, Jensen et al. and Benedikt et al. developed their famous basis sets (aug)pcJ-*n* (*n* = 0–4) [82,95,96] and ccJ-pVXZ (X = D, T, Q, 5) [97], which are suitable for the calculation of the SSCCs involving 1–3 and 1–2 row nuclei, respectively. 

In this paper, we present new compact *J*-oriented basis sets for the selenium atom that have been obtained within the property-energy consistent (PEC) method, introduced in our previous studies [98,99,100]. The PEC method is based on the consistent optimization of all exponents using the Monte Carlo (MC) simulations [101,102,103], with respect to the property under consideration and the total molecular energy. It gives compact basis sets that are capable of providing accuracy comparable to that achieved with the other property-oriented basis sets of larger sizes generated with a standard even-tempered technique [104].

This work has been called for by the needs of compact and accurate basis sets for highly computationally demanding calculations of the spin–spin coupling constants involving selenium in large- and medium-sized selenium compounds. Only a few *J*-oriented basis sets for selenium exist at the moment, and all of them are rather large to be efficiently used in large-scale calculations. These are the acvXz-J (X = 2, 3, 4) [93], the aug-cc-pVTZ-J (the first version) [86], and the aug-cc-pVTZ-J (the second version) [87]. The aug-cc-pVTZ-J (the first version) basis set for selenium was obtained from the energy-optimized uncontracted Dunning’s basis set of triple-zeta quality, aug-cc-pVTZ(uc) (21*s*14*p*10*d*2*f*) [105], by the addition of two tight *s*-type functions and the subsequent application of the contraction scheme based on the molecular orbital coefficients of the selenium hydride, (23*s*14*p*10*d*2*f*) -> [12*s*9*p*6*d*2*f*]. The *f*- and *g*-angular spaces were not considered in the saturation procedure in the mentioned paper. The acvXz-J (X = 2, 3, 4) basis sets [93] were designed from the augmented core-valence Dyall’s basis sets dyall.acvXz (X = 2, 3, 4) [106,107,108] of double- (16*s*12*p*8*d*2*f*), triple- (24*s*17*p*11*d*5*f*1*g*), and quadruple-zeta (31*s*22*p*14*d*6*f*4*g*1*h*) quality by the consecutive even-tempered saturation of the *s*-, *p*-, *d*-, *f*-, and *g*-angular functional spaces. As a result, it was found that the double-zeta quality basis set dyall.acv2z is not complete in the *f*-space for the correct calculation of the selenium SSCCs; i.e., two *f*-functions are not enough. For example, the addition of only one diffuse *f*-function to the *f*-space of dyall.acv2z resulted in the increase of the ^1^*J*(^77^Se,^1^H) of MeSeH molecule by about 5 Hz. Thus, the proposed acvXz-J basis sets were profoundly expanded in the *f*-space as compared to the corresponding dyall.acvXz basis sets. Therefore, the acvXz-J basis sets have the following configurations: [19*s*12*p*8*d*4*f*|11*s*8*p*5*d*4*f*] for X = 2, [27*s*17*p*11*d*5*f*1*g*|14*s*10*p*6*d*5*f*1*g*] for X = 3, and [35*s*22*p*14*d*6*f*4*g*|18*s*12*p*7*d*6*f*4*g*] for X = 4. In this sense, the aug-cc-pVTZ-J for the selenium atom has also been revisited recently [87] because the former version of this basis set contained only two *f*-functions. Thus, the contemporary configuration of the aug-cc-pVTZ-J for the selenium atom is [26*s*16*p*12*d*5*f*|17*s*10*p*7*d*5*f*]. Although the acvXz-J and aug-cc-pVTZ-J (the second version) basis sets are complete enough in all important functional spaces to provide a flexible description of the selenium SSCCs, the problem is that these basis sets are very large. To be more precise, the number of functions for the uncontracted/contracted forms of these basis sets is as follows: 123/88 (acv2z-J), 177/118 (acv3z-J), 249/167 (acv4z-J), and 169/117 (aug-cc-pVTZ-J). On the other hand, we have recently proposed the PEC method [98] for generating property-oriented basis sets that reoptimizes all angular spaces and gives very efficient compact basis sets, providing more accurate results as compared to the property-oriented even-tempered basis sets of similar sizes. Thus, in this paper, we present new pecJ-*n* (*n* = 1, 2) basis sets for the calculation of selenium SSCCs that embody both desirable features: they are moderate in size and provide very accurate results.

## 2. Results and Discussion

### 2.1. Creation of pecJ-n (n = 1, 2) Basis Sets for Selenium

The PEC method [98] consists of the optimization of basis sets in relation to a certain molecular property, provided that the least possible total molecular energy is achieved. Exponents are randomly generated around the starting basis set via Monte Carlo simulations. The generated arrays are verified whether they give the property under interest within a desired diapason or not. In the end, only one set is selected—the one that provides the property value within the desired range and the lowest energy. For the detailed description of the PEC algorithm and its peculiarities as applied to the generation of the *J*-oriented basis sets, we refer the reader to our earlier works presenting the basics of the method [98,99]. Although, it should be mentioned that the PEC method is unique in the sense of dealing with two target functions (the property and the energy) simultaneously. The PEC optimization procedure of the exponents {*ζ_i_*} for the property under consideration with respect to the “ideal” values under the energetic constrain represents a nonlinear problem with multiple solutions. In this case, the PEC optimization procedure can be thought of as approaching the isoline of the “ideal” property value that is formed by the intersection of the “ideal” property plane with the property surface representing the multi-argument function of the varying exponents *f* (*ζ*_1_, *ζ*_2_, …, *ζ_n_*), both determined in the multidimensional exponential space. All points of this isoline give the same “ideal” value of property but different molecular energies. The PEC method is aimed at selecting the basis set that provides the lowest molecular energy among those which belong to the “ideal” isoline, since the lower the energy, the better the description of the molecular wave-function. The optimization problem in the multidimensional exponential space with multiple solutions can hardly be solved using standard procedures based on the directed search, like numerical Newton-like methods [109]. This is connected with the natural limitations of such techniques due to the fact that they are aimed at finding a single extremum of a property in the vicinity of the starting point. In the case of Newton-type optimization, one can imagine the process as a gradual ascending or descending to a certain single point. In that way, such algorithms lapse the other solutions, unless initiated many times from different starting guesses, and, even if they were started from various guesses, they would find only a limited number of the solutions. Based on this reasoning, one can conclude that a conventional directed Newton-like optimization is not fully suitable for the problem with multiple solutions; in this sense, the PEC method is unique and totally justified for generating the *J*-oriented basis sets. 

In this work, all optimizations of exponents were carried out using the SOPPA(CCSD) method for SSCC calculations and the CCSD method for the energy calculations. We used two fitting molecules, SeH_2_ and Se=C, in which the FC contributions to ^1^*J*(^77^Se,^1^H) and ^1^*J*(^77^Se,^13^C) SSCCs were considered as the arguments of the target function. The target function that was minimized represents the mean absolute error of these *J*_FC_ against their “ideal” values:

(1) $$\tilde{\Delta }=\frac{1}{2}\sum_{i=1}^{2}\left|{\tilde{J}}_{FC,i}-J_{FC,i}^{ideal}\right|\to \min$$

This minimization was performed under the energetic constrain: $\overset{2}{\underset{n=1}{\sum }}{\tilde{E}}_{n}\to \min$, which guarantees that the least possible total molecular energy of two molecules is achieved. The energy tolerance threshold was set to 10^−4^ Hartree. The optimization that involves two fitting molecules provides more robustness of the generated basis sets towards the diversity of the electronic systems as compared to the basis sets obtained with only one fitting molecule. 

The “ideal” values were evaluated at the SOPPA(CCSD) level using the extended dyall.aae4z basis set (dyall.aae4z^+^) on all atoms in both fitting molecules. The dyall.aae4z^+^ was obtained by adding 3, 2, and 1 tight *s*-functions to the dyall.aae4z basis set for hydrogen, carbon, and the selenium atom, respectively, in the even-tempered manner. Accordingly, the corresponding configurations of the dyall.aae4z^+^ basis set for these atoms are as follows: (15*s*4*p*3*d*2*f*), (21*s*11*p*6*d*4*f*2*g*), and (32*s*22*p*14*d*9*f*5*g*1*h*). In this respect, dyall.aae4z^+^ provides overwhelming flexibility in all angular spaces, including the most important tight *s*-region to gain the values of SSCCs that are very close to the CBS limit. Thus, JFC,1ideal=JFCideal1Se77,H1=74.65 Hz and JFC,2ideal=JFCideal1Se77,C13=−154.25 Hz. It is interesting to note that the ideal values for the FC contributions to one-bond ^77^Se-^1^H and ^77^Se-^13^C SSCCs are of different signs. The sign of the *J*_FC_(A,B) can be deduced from the signs of the nuclear magnetogyric ratios of the coupled nuclei (γ_A_, γ_B_) and that of the reduced SSCC, *K*_FC_(A,B), in accordance with the following relationship: *J*_FC_(A,B) = (h/4π^2^)∙(γ_A_γ_B_)∙*K*_FC_(A,B). As the magnetogyric ratios of the isotopes ^1^H, ^13^C, and ^77^Se are of the same positive sign, the difference in the sign of the ^1^*J*_FC_(^77^Se,^1^H) and ^1^*J*_FC_(^77^Se,^13^C) stems from different signs of the FC contributions to the corresponding reduced SSCCs. In this respect, the latter is totally determined by the details of the electronic structure of the considered molecules and can be deduced based on the fundamental rules proposed by Gil and von Philipsborn [110]. The authors proved that, if one of the coupled nuclei has lone electron pair(s) (LEP(s)), they always give the contributions of a negative sign to ^1^*K*_FC_(A,B). As a consequence, the removal of a lone electron pair, namely by protonation, alkylation, oxide formation, or complexation, leads to an increase in ^1^*K*_FC_(A,B). The negative contributions from LEP(s) (^1^*K*_FC_^LP^) compete with the always positive contributions from the bonding orbital between coupled nuclei (^1^*K*_FC_^BD^) [111]. In the case of the SeH_2_ molecule, the ^1^*K*_FC_^BD^ is very large [111] and, apparently, is not quite suppressed by the competing ^1^*K*_FC_^LP^, giving (in total with minor rest contributions) a relatively small positive ^1^*K*_FC_(Se,H) [43], while for most ^1^*J*(Se,C) SSCCs, the FC contribution has a negative sign [32,43], which can be related to the total domination of the negative ^1^*K*_FC_^LP^ over the positive ^1^*K*_FC_^BD^. 

**Figure 1:**
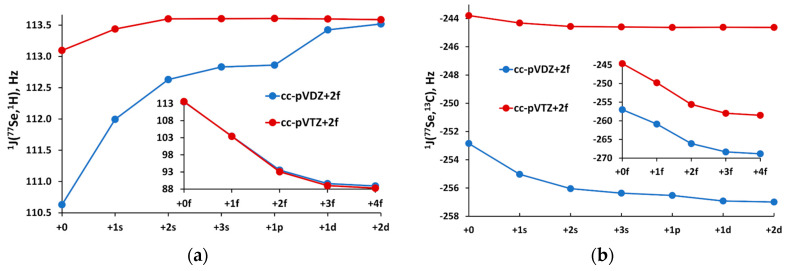
Convergence of the one-bond SSCCs of SeH_2_ and Se=C upon saturation of cc-pVDZ+2*f* and cc-pVTZ+2*f* basis sets set on selenium atom: (**a**) ^1^*J*(^77^Se,^1^H); (**b**) ^1^*J*(^77^Se,^13^C).

It was shown earlier [87,93] that the expansion of the *f*-angular space of a standard energy-optimized basis set of double-zeta quality leads to a dramatic change in the one-bond SSCCs involving selenium and other NMR-active half spin nuclei. A weaker but no less important sensitivity of the selenium SSCCs has been found when varying the tight *s*-region. Therefore, before starting the basis set optimization process, we carried out the investigation of the sensitivity of ^1^*J*(^77^Se,^1^H) of SeH_2_ and ^1^*J*(^77^Se,^13^C) of Se=C to the expansion of different angular spaces of basis sets of double- and triple-zeta quality on the selenium atom. For that purpose, we have set the uncontracted pecJ-1 or pecJ-2 basis sets on hydrogen and carbon atoms [98], while the cc-pVDZ+2*f* or cc-pVTZ+2*f* basis sets were set on the selenium atom. The cc-pVDZ+2*f* represents the uncontracted cc-pVDZ basis set [105] augmented with both *f-*functions of aug-cc-pVTZ basis set [105], while the cc-pVTZ+2*f* is the uncontracted cc-pVTZ basis set [105], whose single *f-*function was replaced with two *f-*functions of the aug-cc-pVTZ basis set. These basis sets were considered the starting sets for further expansion. The behaviors of the ^1^*J*(^77^Se,^1^H) and ^1^*J*(^77^Se,^13^C) SSCCs upon the saturation of the cc-pVDZ+2*f* or cc-pVTZ+2*f* basis sets on selenium atom are shown in Figure 1a,b.

The saturation of basis sets on the selenium atom was carried out in the tight region of each angular space by means of applying the geometrical progression or even-tempered recurrent ratio *ζ_i_* = *αβ_i_*_,_ with *α* representing the largest exponent *ζ_n_* in the original functional set and *β* being the ratio of two largest exponents, *β = ζ_n_/ζ_n_*_−1_. The saturation of basis sets has been made in the consecutive manner, that is, we passed to the saturation of the next angular space only if the current space was totally saturated and provided the converged value. 

From Figure 1a,b, one can see that the addition of two tight *s*-functions to both cc-pVDZ+2*f* and cc-pVTZ+2*f* basis sets provides the converged values in both cases. Thus, two *s*-functions have been added to their initial configurations. The modifications made in the *p*-space did not cause any effect, while the expansion of the *d*-space slightly affected both SSCCs only in the case the of cc-pVDZ+2*f* basis set. Thus, it was decided to add one *d*-function to the cc-pVDZ+2*f* basis set and to remove one *d*-function from the cc-pVTZ+2*f* basis set, for the sake of lowering the least needed size of the *d*-space in the latter case. The expansion of the *f*-region resulted in very substantial changes in both SSCCs calculated with both double- and triple-zeta basis sets. As can be seen from Figure 1, the decrease of the ^1^*J*(^77^Se,^1^H) and ^1^*J*(^77^Se,^13^C) upon adding four additional tight functions to the *f*-space of both basis sets is about 25 and 12–14 Hz, respectively. Figure 1 tells us that the least needed number of additional *f*-functions to the original two functions is three in all cases, thus giving five *f*-functions in total. In that way, five *f*-functions provide sufficiently converged values within the *f*-space. However, we decided to deal with only four and three *f*-functions in the *f*-spaces of the pecJ-2 and pecJ-1 basis sets, respectively. This seemingly controversial decision can be explained by the very essence of our PEC method. This method is not a mere even-tempered augmentation of the basis set but an optimization of the exponents that implies dealing with a fewer number of exponents providing the same or even better accuracy than the larger even-tempered basis set. Thus, leaning on our experience of the PEC method, three additional even-tempered exponents can be replaced with one and two optimized exponents for the double- and triple-zeta levels, respectively, resulting in three and four *f*-functions in total. That is enough for the correct representation of the first polarization shell. This also can be thought of as an explanation of our decision of removing one *d*-function in the final configuration of the pecJ-2 basis set. We also decided to introduce one *g*-function to improve the description of the second polarization shell of the selenium atom. The resulting configurations and the modifications made are represented in Table 1.

Thus, having the resulting configurations for the pecJ-*n* basis sets, we have set the obtained trial basis sets on the selenium atom and pecJ-1 or pecJ-2 basis sets on the hydrogens and carbon atoms in the fitting molecules SeH_2_ and Se=C, and commenced the PEC optimization of the exponents. It is worth mentioning once again that, in the current case, we considered only the FC terms of the selenium SSCCs. In this sense, it could be said that we have developed the pecJ-*n* basis sets for the FC-dominating selenium SSCCs, covering by that the majority of cases [32,47,48,50,112]. It is also very important that all exponents in all shells were varied during the optimization process, which is different from what was conducted in our first work on the PEC method [98], where for the nonhydrogen atoms of the second period, only the *s*-, *d*-, and *f*-shells were varied to converge the target FC term to the ideal value. The final deviations of the FC contributions to the SSCCs of both fitting molecules from their ideal values amounted to ca. 0.01 Hz for both pecJ-1 and pecJ-2 basis sets. The resulting optimized exponents of the pecJ-1 and pecJ-2 basis sets can be found in Tables S2–S5 of Supplementary Materials, where the final selenium pecJ-*n* basis sets are presented in the format of the Dalton [113] and CFOUR [114] programs.

To reduce the sizes of the obtained uncontracted basis sets (for that we will use the notation “(uc)” throughout the text), pecJ-*n*(uc), we have applied a general contraction scheme [115]. However, this time, it was not a mere gain of the contraction coefficients from the molecular energy Self-Consistent Field (SCF) calculations of the simplest hydrides but was the application of the PEC algorithm to minimize the contraction error, providing the least possible molecular energy. In more detail, to obtain the contraction coefficients for the selenium pecJ-*n* basis sets, we consecutively (in relation to shells) minimized the sum of the absolute differences between the values of the ^1^*J*(^77^Se,^1^H) of SeH_2_ and ^1^*J*(^77^Se,^13^C) of Se=C (this time, it was the total values consisting of the four Ramsey contributions) and the corresponding contemporary reference values. The pecJ-1 or pecJ-2 basis sets were set on the hydrogen and carbon atoms, depending on the cardinal number of the basis set used on the selenium atom. This routine can be thought of as the following:

1. The PEC optimization of the contraction coefficients for the *s*-shell with respect to the target function: $\tilde{\Delta }=\frac{1}{2}\sum_{i=1}^{2}\left|{\tilde{J}}_{i}-J_{i}^{ref}\right|$ is performed. The ${\tilde{J}}_{i}$ are the values obtained with chosen *s*-contraction pattern with the varying contraction coefficients, and $J_{i}^{ref}$ are the values obtained with the fully uncontracted pecJ-*n*(uc) basis set on selenium. When the optimization procedure is completed, the reference values $J_{i}^{ref}$ are redefined: the new ones are obtained with the modified pecJ-*n* basis set on selenium, in which the *s*-shell is contracted with the optimized coefficients, and the remaining shells are uncontracted;
2. The PEC optimization of the contraction coefficients for the *p*-shell starts; the reference values are those that were recalculated in the previous step. When the optimized coefficients for the *p*-shell are obtained, the $J_{i}^{ref}$ are redefined once again: the new ones are calculated with the pecJ-*n* basis set, in which the *s*-shell is contracted with the coefficients obtained in the first step and *p*-shell is contracted with the newly optimized coefficients, while the remaining shells are kept uncontracted;
3. The PEC optimization of the contraction coefficients for the *d*-shell starts; the reference values are those that were recalculated in the previous step. In the end, we arrive at the final pecJ-*n* basis set with the contracted *s*-, *p*-, and *d*-shells and uncontracted *f*-shell, plus 1*g* function in the pecJ-2 basis set.

During this algorithm, the contraction patterns with different contraction depths were considered for each shell. A decision for the choice of the number of contracted functions was made based on the resulting contraction error Δ for each step. Table 2 shows different contraction patterns with the final averaged absolute errors (Δ) in relation to the current reference values.

The final configurations of the pecJ-*n* basis sets for the selenium atom together with the mean absolute percentage errors (ε), which they provide at the SOPPA(CCSD) level against the values obtained with the totally uncontracted pecJ-*n*(uc) basis set used on the selenium atom, are compiled in Table 3. The final contraction coefficients for the pecJ-*n* basis sets are presented in Tables S2–S5 of Supplementary Materials.

It is worth mentioning that the contracted aug-cc-pVTZ-J basis set for the selenium atom has the configuration [17*s*10*p*7*d*5*f*], thus providing the *N*_bas_ of 117. Our contracted second-level basis set, pecJ-2, has a pronounced benefit in size as compared to the aug-cc-pVTZ-J basis set, being 16 basis set functions fewer than the latter. If we recall the formal computational scaling [62] for the most popular high-quality computational methods applied to the calculation of SSCCs, namely *N*^5^ for SOPPA, *N*^5^ for SOPPA(CC2), *N*^6^ for SOPPA(CCSD), *N*^6^ for CCSD, and *N*^4^ for DFT, with *N* being the total number of basis set functions participating in the calculation, the additional 16 functions on selenium result in a significant increasing of the operations needed to evaluate characteristic operators in a molecular orbital basis. To roughly exemplify the operational costs provided by the pecJ-*n* and aug-cc-pVTZ-J basis sets, we show the assessment of *N^k^* (with *k* being the scaling factors of different methods) for the simplest selenium hydride, SeH_2_; see Table 4. We set the basis set of the same type on the hydrogen atom.

From Table 4, it can be seen that the pecJ-*n* basis sets are beneficial compared to the aug-cc-pVTZ-J basis set. For example, for the most popular SOPPA and CCSD methods, the calculations with the pecJ-1 and pecJ-2 basis sets are, respectively, ten and two times less computationally demanding than that with the aug-cc-pVTZ-J basis set. 

### 2.2. Testing New Basis Sets

In this section, we demonstrate the accuracy provided by our new basis sets against the experiment. All couplings were calculated at the CCSD level of theory, taking into account vibrational, solvent, and relativistic corrections to be properly compared with the experimental data. Overall, we calculated 13 selenium SSCCs of different types in seven representative molecules. The basic CCSD values were calculated with the pecJ-*n* and aug-cc-pVTZ-J basis sets being set on all atoms with the exclusion of chlorine; for the chlorine, atom we used the Dunning’s correlation consistent basis sets of different qualities depending on the basis sets used on the rest of atoms, namely, the cc-pVDZ on Cl with the pecJ-1 on the rest, and cc-pVTZ on Cl with the pecJ-2 or aug-cc-pVTZ-J on the rest. All three types of corrections were calculated using the SVWN5 exchange-correlation functional [116,117]. The SVWN5 represents the local density approximation (LDA) [118,119], in which the exchange is uniquely defined analytically in the form of the exchange energy of a homogeneous electron gas, while its correlation term is defined through several parameterizations, mostly relying on the highly accurate quantum Monte Carlo simulations. We have chosen this function based on the fact that the LDA model is particularly stable towards triplet instabilities [120], reflecting a balanced description of exchange and correlation. The latter is very important for the calculation of triplet excitation properties such as FC and SD contributions to the spin–spin coupling constants. 

The solvent corrections to SSCCs were calculated as the differences between their values obtained in the gas and liquid phases. The IEF-PCM scheme [121,122] for a particular solvent (specified in accordance with the experimental data) was used for each compound in the liquid phase simulation. In the calculations of the solvent corrections, the same basis set schemes as those applied in the CCSD calculations were used.

The vibrational corrections to the SSCCs were calculated at zero temperature (zero-point vibrational corrections, ZPVC) within the vibrational second-order perturbation theory (VPT2) [123] as applied in combination with the effective geometry approach of Ruud et al. [124]. The effective geometry represents the vibrationally averaged molecular geometry to the second-order in perturbation theory (involving the cubic force-field tensor). Thus, by means of calculating the property at the effective geometry, one partially takes into account the contribution to a vibrationally averaged property due to the anharmonicity of the potential, while the inclusion of the contribution from the averaging of the molecular property over the harmonic oscillator requires the additional step where the second derivative of the property surface is calculated at the effective geometry. In all vibrational calculations, we used the pecJ-1 basis set on all atoms except for the chlorine atom, on which the cc-pVDZ basis set was used.

The relativistic corrections to coupling constants were evaluated as the differences between the SSCCs obtained using the relativistic four-component Dirac–Kohn–Sham–Hamiltonian and those obtained within the “10c limit scheme”. The “10c limit scheme” implies the increase of the speed of light by 10 times in the relativistic four-component calculations, resulting in a sufficiently accurate approximation of nonrelativistic values. 

The “10c limit scheme” is now a widely used approximation [24,25,93,94,125,126,127,128,129] that is applied to exclude the basis set inequivalence in passing from the four-component relativistic to the one-component nonrelativistic framework. The convergences of the selenium SSCCs with the increasing of the speed of light are shown in Figures S1 and S2 in the Supplementary Materials for the examples of ^1^*J*(Se,H) in SeH_2_ and ^1^*J*(Se,C) in Se=C, respectively, calculated at the four-component DFT(SVWN5)//pecJ-2(uc) level. The speed of light was increased from one (totally relativistic calculation) to ten (“10c limit scheme”) times. It follows that, for both molecules, the convergence within ca. 0.1–0.2 Hz already appears at the increasing of the speed of light by seven to eight times. Thus, the “10c limit scheme” can be regarded as appropriate for the calculations of the selenium SSCCs considered in this work. 

In the four-component calculations, the same basis sets as in the CCSD calculations were used, though applied in the uncontracted form. The four-component calculations were performed under the unrestricted kinetic balance condition (UKB) [130,131].

The results are compiled in Table 5 and Table 6, with the former presenting the roles of four Ramsey contributions to total CCSD values of SSCCs and the latter showing the comparison of the corrected CCSD values with the experiment.

As can be seen from Table 5, the FC contribution by far dominates the other three contributions for most of the considered SSCCs. Only in the case of Se-P SSCCs are the PSO contributions significant on an absolute scale, though, as compared to the FC terms of the Se-P SSCCs, these are not so significant, being only about 20% of the magnitude of the FC terms. It is also worth mentioning that in some one-bond Se-C and Se-H SSCCs, the SD contribution is noticeable on an absolute scale (for example, in compounds **2**, **5**, **7**), though being substantially inferior to the FC terms. 

**Figure 2:**
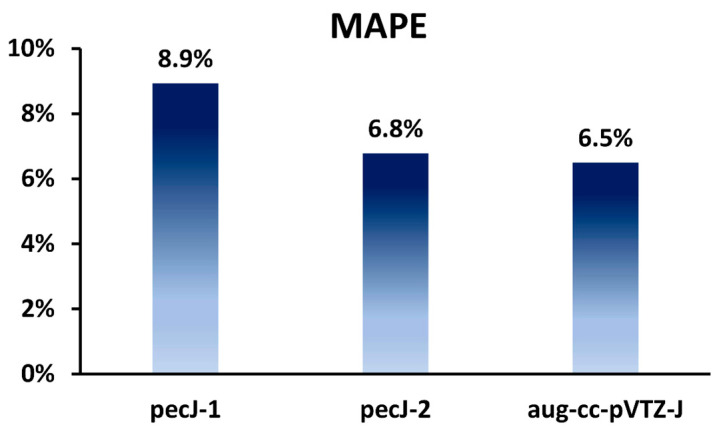
Mean absolute percentage errors (MAPEs) for all computed SSCCs involving selenium against experiment depending on the basis set used.

As can be seen from Table 6, the agreement of total theoretical values obtained with introduced basis sets with the experimental data is rather good. The mean absolute percentage errors (MAPEs) evaluated for all final theoretical selenium SSCCs against the experiment are presented in Figure 2. 

From Figure 2, one can see that the total accuracy provided by the pecJ-2 basis set (MAPE = 6.8%) is practically the same as that provided by a rather larger aug-cc-pVTZ-J basis set (MAPE = 6.5%). In terms of formal operational costs, for the most popular SOPPA or CCSD methods, we can arrive at a 50% reduction (as was roughly estimated above in the example of selenium hydride) of the number of operations without any noticeable loss of accuracy by using the pecJ-2 basis set instead of the aug-cc-pVTZ-J basis set. Meanwhile, the calculations with the pecJ-1 basis set are several times less computationally demanding than those with the pecJ-2 basis set, and the accuracy is only slightly (MAPE = 8.9 vs. 6.8%) inferior to that provided by the pecJ-2 basis set. Thus, we can conclude here that our compact pecJ-1 basis set may be very useful in large-scale calculations or in very demanding calculations like those with highly correlated methods involving triple- or higher excitations or in the problem of vibrational averaging. 

## 3. Materials and Methods

Geometry optimizations were carried out at the DFT level of theory with the Minnesota M06-2X exchange-correlation functional [137] using the pc-3 basis set [138,139,140,141]. Media effects were taken into account within the IEF-PCM solvation model when optimizing the geometrical parameters for the testing compounds **1**–**7**, while the optimization of the geometry of both fitting molecules, SeH_2_ and Se=C, has been performed in the gas phase. All optimizations were performed in the Gaussian program [142]. Final equilibrium geometries are presented in Table S1 of Supplementary Materials. The SOPPA(CCSD) and CCSD calculations of SSCCs were carried out in the Dalton [113] and CFOUR [114] programs, respectively. Solvent and vibrational corrections to SSCCs were calculated using the Dalton program. Relativistic values were calculated within the DIRAC program [143]. 

## 4. Conclusions

We presented new compact and accurate pecJ-*n* (*n* = 1, 2) basis sets for the selenium atom purposed for the quantum–chemical calculations of NMR spin–spin coupling constants involving selenium nuclei. These basis sets were obtained with the property-energy consistent method, which is efficient in generating compact property-oriented basis sets due to its peculiarity to fully reoptimize all angular spaces of the trial basis sets. In this way, the final PEC-generated basis sets are significantly smaller than the other specialized basis sets of the same zeta-quality obtained with the even-tempered technique. Thus, new pecJ-1 and pecJ-2 basis sets consist of only 78 and 101 basis functions, respectively. This gives a significant benefit as compared to the other existing *J*-oriented selenium basis sets, acvXz-J (X = 2, 3, 4) and aug-cc-pVTZ-J, with the total number of functions being as much as 88, 118, 167, and 117, respectively. In particular, for the SeH_2_ molecule, the operational costs of the SOPPA and CCSD calculations can be said to be significantly reduced by using the pecJ-1 and pecJ-2 basis sets by approximately ten and two times, respectively, as compared to the costs of the calculations with the aug-cc-pVTZ-J basis set. 

New basis sets were tested on the CCSD calculations of thirteen SSCCs involving selenium in the representative series of seven molecules carried out with taking into account relativistic, solvent, and vibrational corrections. The comparison with the experiment revealed that the accuracy of the results obtained with a compact pecJ-2 basis set is almost the same as that provided by an essentially larger basis set, aug-cc-pVTZ-J (the MAPEs are 6.8% and 6.5% for the former and the latter, respectively), while the accuracy achieved with a significantly smaller basis set, pecJ-1, is only slightly inferior (MAPE = 8.9%) to the accuracy provided by the pecJ-2 basis set. Overall, we recommend resorting to the pecJ-2 basis set in large-scale calculations of selenium SSCCs; this would provide accuracy comparable to that of the aug-cc-pVTZ-J basis set and give significant CPU time savings. The pecJ-1 can be recommended for highly demanding calculations, such as those involving the coupled cluster methods of higher hierarchy that treat triple- and higher excitations or in the problem of vibrational averaging.

## Figures and Tables

**Table 1:** Modifications in original configuration of cc-pVXZ+2*f* (X = D, T).

Starting Configuration	Total Modification	Resulting Configuration
cc-pVDZ+2*f*: (14*s*11*p*6*d*2*f*)	+2*s*, +0*p*, +1*d*, +1*f*	pecJ-1: (16*s*11*p*7*d*3*f*)
cc-pVTZ+2*f*: (20*s*13*p*9*d*2*f*)	+2*s*, +0*p*, –1*d*, +2*f*, +1*g*	pecJ-2: (22*s*13*p*8*d*4*f*1*g*)

**Table 2:** Contraction patterns with varying depths ^1^.

Step	Basis Set	Contraction Pattern	Detailed Contraction Pattern	Final Contraction Error Δ (in Relation to Current Reference Values), in Hz
Step 1 (*s*-shell)	pecJ-1	16s → 10*s*	(8,8,1,1,1,1,1,1,1,1)	0.133
		**16*s*** → **11*s***	**(7,7,1,1,1,1,1,1,1,1,1)**	**0.024**
		16s → 12*s*	(6,6,1,1,1,1,1,1,1,1,1,1)	0.011
	pecJ-2	22s → 14*s*	(10,10,1,1,1,1,1,1,1,1,1,1,1,1)	0.133
		**22*s*** → **15*s***	**(9,9,1,1,1,1,1,1,1,1,1,1,1,1,1)**	**0.091**
		22s → 16*s*	(8,8,1,1,1,1,1,1,1,1,1,1,1,1,1,1)	0.077
Step 2 (*p*-shell)	pecJ-1	11p → 6*p*	(7,7,1,1,1,1)	1.080
		**11*p*** → **7*p***	**(6,6,1,1,1,1,1)**	**0.095**
		11p → 8*p*	(5,5,1,1,1,1,1,1)	0.076
	pecJ-2	13p → 7*p*	(8,8,1,1,1,1,1)	0.998
		**13*p*** → **8*p***	**(7,7,1,1,1,1,1,1)**	**0.047**
		13p → 9*p*	(6,6,1,1,1,1,1,1,1)	0.032
Step 3 (*d*-shell)	pecJ-1	7d → 4*d*	(4,1,1,1)	0.768
		**7*d*** → **5*d***	**(3,1,1,1,1)**	**0.123**
		7d → 6*d*	(2,1,1,1,1,1)	0.098
	pecJ-2	8d → 4*d*	(5,1,1,1)	0.676
		**8*d*** → **5*d***	**(4,1,1,1,1)**	**0.127**
		8d → 6*d*	(3,1,1,1,1,1)	0.124

^1^ Bold face indicates the chosen patterns.

**Table 3:** Final configurations of pecJ-*n* (*n* = 1, 2) for selenium atom.

Final Configuration	*N*_bas_/*N*_bas(uc)_	*ε*_1/2_, in % ^1^
pecJ-1: [16*s*11*p*7*d*3*f*|11*s*7*p*5*d*3*f*]	78/105	*ε*_1_ = 0.35; *ε*_2_ = 0.08
pecJ-2: [22*s*13*p*8*d*4*f*1*g*|15*s*8*p*5*d*4*f*1*g*]	101/138	*ε*_1_ = 0.18; *ε*_2_ = 0.04

^1^*ε*_1_ and *ε*_2_ are the relative contraction errors of the pecJ-*n* basis sets calculated for ^1^*J*(^77^Se,^1^H) in SeH_2_ and ^1^*J*(^77^Se,^13^C) in Se=C calculated as: *ε*_1_ = (|^1^*J*(^77^Se,^1^H) [pecJ-*n*] − ^1^*J*(^77^Se,^1^H) [pecJ-*n*(uc)] |/|^1^*J*(^77^Se,^1^H) [pecJ-*n*(uc)]|)·100%, *ε*_2_ =(|^1^*J*(^77^Se,^13^C) [pecJ-*n*] − ^1^*J*(^77^Se,^13^C) [pecJ-*n*(uc)] |/|^1^*J*(^77^Se,^13^C) [pecJ-*n*(uc)]|) × 100%, respectively. Previously developed contracted pecJ-*n* basis sets were used on the hydrogen and carbon atoms.

**Table 4:** Formal operational costs of different methods provided by the pecJ-*n* and aug-cc-pVTZ-J basis sets for SeH_2_ ^1^.

Basis Set	*N*	*N*^4^ (DFT, RPA)	*N*^5^ (SOPPA, SOPPA (CC2))	*N*^6^ (SOPPA(CCSD), CCSD)	*N*^8^ (CCSDT)
pecJ-1	78(Se) + 2 × 11(H) = 100	1.00 × 10^8^, *R* = 0.16	1.00 × 10^10^, *R* = 0.10	1.00 × 10^12^, *R* = 0.07	1.00 × 10^16^, *R* = 0.03
pecJ-2	101(Se) + 2 × 20(H) = 141	3.95 × 10^8^, *R* = 0.65	5.57 × 10^10^, *R* = 0.58	7.86 × 10^12^, *R* = 0.52	1.56 × 10^17^, *R* = 0.42
aug-cc-pVTZ-J	117(Se) + 2 × 20(H) = 157	6.08 × 10^8^ *R* = 1.00	9.54 × 10^10^ *R* = 1.00	1.50 × 10^13^ *R* = 1.00	3.69 × 10^17^, *R* = 1.00

^1^ *R* = (*N*_pec-J-*n*_/*N_aug-cc-pVTZ-J_*)*^k^* represents the ratio of the operational costs of methods with a particular scaling factor *k* provided by pecJ-*n* and aug-cc-pVTZ-J basis sets. For the aug-cc-pVTZ-J basis set, this factor is equal to 1.

**Table 5:** Different contributions to SSCCs ^1^ involving selenium in compounds **1**–**7** calculated at the CCSD level of theory with pecJ-*n* (*n* = 1, 2) basis sets.

Molecule	SSCC	Basis Set	*J*_FC_	*J*_SD_	*J*_PSO_	*J*_DSO_	Total *J*_CCSD_
Me_2_Se_2_ (**1**)	^1^*J*(^77^Se,^13^C)	pecJ-1	−77.21	11.26	6.61	0.09	−59.25
		pecJ-2	−81.26	12.32	6.74	0.09	−62.11
	^2^*J*(^77^Se,^13^C)	pecJ-1	4.13	1.60	2.00	−0.02	7.71
		pecJ-2	3.34	1.57	1.77	−0.02	6.66
MeSe-C≡N (**2**)	^1^*J*(^77^Se,^13^C*_sp_*_3_)	pecJ-1	−57.23	12.17	9.32	0.05	−35.69
		pecJ-2	−60.95	12.53	9.25	0.05	−39.12
	^1^*J*(^77^Se,^13^C*_sp_*)	pecJ-1	−173.73	1.96	−6.90	0.10	−178.57
		pecJ-2	−182.11	2.21	−6.17	0.10	−185.97
MeSeH (**3**)	^1^*J*(^77^Se,^13^C)	pecJ-1	−60.72	12.25	11.77	0.04	−36.66
		pecJ-2	−64.61	12.68	12.00	0.04	−39.89
Me_2_P(Se)Cl (**4**)	^1^*J*(^77^Se,^31^P)	pecJ-1	−688.03	−1.55	−152.96	0.12	−842.42
		pecJ-2	−700.90	−0.74	−151.21	0.12	−852.73
C_4_H_4_Se (**5**)	^2^*J*(^77^Se,^1^H)	pecJ-1	47.50	−0.15	−4.61	−0.21	42.53
		pecJ-2	48.97	−0.16	−4.92	−0.21	43.68
	^3^*J*(^77^Se,^1^H)	pecJ-1	8.59	0.26	0.56	−0.36	9.05
		pecJ-2	8.74	0.45	0.38	−0.36	9.21
	^1^*J*(^77^Se,^13^C)	pecJ-1	−59.91	2.31	−25.79	0.09	−83.3
		pecJ-2	−65.34	2.16	−26.53	0.09	−89.62
	^2^*J*(^77^Se,^13^C)	pecJ-1	−1.83	2.92	−1.20	−0.02	−0.13
		pecJ-2	−2.31	3.7	−1.51	−0.02	−0.14
SePMe_3_ (**6**)	^1^*J*(^77^Se,^31^P)	pecJ-1	−619.15	4.24	−108.92	0.1	−723.73
		pecJ-2	−621.69	6.15	−104.12	0.1	−719.56
SiH_3_SeH (**7**)	^1^*J*(^77^Se,^1^H)	pecJ-1	47.12	−0.48	23.12	0.12	69.88
		pecJ-2	48.41	−0.04	22.5	0.11	70.98
	^2^*J*(^77^Se,^1^H)	pecJ-1	15.81	−0.25	0.13	−0.18	15.51
		pecJ-2	15.91	−0.25	0.13	−0.18	15.61

^1^ All values are given in Hz.

**Table 6:** Testing pecJ-*n* (*n* = 1, 2) basis sets on the calculation of SSCCs ^1^ involving selenium in compounds **1**–**7** against experiment ^2^.

Molecule	SSCC	Basis Set	*J*_CCSD_	Δ_rel_^3^	Δ_solv_^3^	Δ_vib_^3^	*J*_tot_	*J*_exp_
Me_2_Se_2_ (**1**)	^1^*J*(^77^Se,^13^C)	pecJ-1	−59.25	−7.02	1.51	−0.9	−65.66	(−)73.87 ± 0.26
		pecJ-2	−62.11	−5.48	1.56	−0.9	−66.93	
		aug-cc-pVTZ-J	−63.56	−4.51	1.54	−0.9	−67.43	
	^2^*J*(^77^Se,^13^C)	pecJ-1	7.71	−0.29	0.24	−0.11	7.55	7.47 ± 0.26
		pecJ-2	6.66	0.36	0.29	−0.11	7.20	
		aug-cc-pVTZ-J	6.55	−0.64	0.28	−0.11	6.08	
MeSe-C≡N (**2**)	^1^*J*(^77^Se,^13^C*_sp_*_3_)	pecJ-1	−35.69	−5.76	0.24	−1.00	−42.21	(−)52.3
		pecJ-2	−39.12	−6.46	0.24	−1.00	−46.34	
		aug-cc-pVTZ-J	−40.74	−5.49	0.24	−1.00	−46.99	
	^1^*J*(^77^Se,^13^C*_sp_*)	pecJ-1	−178.57	−37.39	−5.84	−3.22	−225.02	(−)237.8
		pecJ-2	−185.97	−37.45	−5.05	−3.22	−231.69	
		aug-cc-pVTZ-J	−188.71	−34.50	−5.28	−3.22	−231.71	
MeSeH (**3**)	^1^*J*(^77^Se,^13^C)	pecJ-1	−36.66	−5.69	2.30	−1.90	−41.95	(−)48.26 ± 0.26
		pecJ-2	−39.89	−6.31	2.39	−1.90	−45.71	
		aug-cc-pVTZ-J	−41.26	−5.19	2.32	−1.90	−46.03	
Me_2_P(Se)Cl (**4**)	^1^*J*(^77^Se,^31^P)	pecJ-1	−842.42	−49.43	11.82	−4.08	−884.11	(−)838.4
		pecJ-2	−852.73	−27.01	14.27	−4.08	−869.55	
		aug-cc-pVTZ-J	−861.31	−28.44	14.12	−4.08	−879.71	
C_4_H_4_Se (**5**)	^2^*J*(^77^Se,^1^H)	pecJ-1	42.53	3.73	0.10	−0.35	46.01	47.6
		pecJ-2	43.68	3.97	0.21	−0.35	47.51	
		aug-cc-pVTZ-J	44.46	3.90	0.19	−0.35	48.20	
	^3^*J*(^77^Se,^1^H)	pecJ-1	9.05	−0.32	0.23	−0.32	8.64	9.4
		pecJ-2	9.21	−0.32	0.33	−0.32	8.90	
		aug-cc-pVTZ-J	9.53	−0.37	0.32	−0.32	9.16	
	^1^*J*(^77^Se,^13^C)	pecJ-1	−83.30	−16.51	0.27	−2.58	−102.12	(−)113.5
		pecJ-2	−89.62	−17.61	0.62	−2.58	−109.19	
		aug-cc-pVTZ-J	−89.18	−17.95	0.60	−2.58	−109.11	
	^2^*J*(^77^Se,^13^C)	pecJ-1	−0.13	−1.50	−0.37	0.19	−1.81	(−)2.7
		pecJ-2	−0.14	−1.59	−0.37	0.19	−1.91	
		aug-cc-pVTZ-J	−1.21	−1.65	−0.38	0.19	−3.05	
SePMe_3_ (**6**)	^1^*J*(^77^Se,^31^P)	pecJ-1	−723.73	−56.56	25.26	−7.45	−762.48	(−)720.0
		pecJ-2	−719.56	−35.36	27.87	−7.45	−734.50	
		aug-cc-pVTZ-J	−725.09	−44.01	27.49	−7.45	−749.06	
SiH_3_SeH (**7**)	^1^*J*(^77^Se,^1^H)	pecJ-1	69.88	−29.49	5.95	4.59	50.93	51.0 ± 0.1
		pecJ-2	70.98	−26.21	6.96	4.59	56.32	
		aug-cc-pVTZ-J	70.83	−26.72	6.52	4.59	55.22	
	^2^*J*(^77^Se,^1^H)	pecJ-1	15.51	0.10	−0.09	−0.16	15.36	15.4 ± 0.2
		pecJ-2	15.61	−0.24	0.01	−0.16	15.22	
		aug-cc-pVTZ-J	15.59	−0.28	−0.04	−0.16	15.11	

^1^ All values are given in Hz. ^2^ Experimental values were taken from different sources: **1**, **3**—[132]; **2**—[133]; **4**—[134]; **5**—[93]; **6**—[135]; **7**—[136]. ^3^ All corrections were calculated at the DFT(SVWN5) level of theory.

## Data Availability

Not applicable.

## Supplementary Materials

The following supporting information can be downloaded at: https://www.mdpi.com/article/10.3390/ijms24097841/s1.
