# Supplementary material for: New pecJ-n (n = 1, 2) Basis Sets for Selenium Atom Purposed for the Calculations of NMR Spin–Spin Coupling Constants Involving Selenium
Source: Int J Mol Sci. 2023 Apr 25;24(9):7841. doi: 10.3390/ijms24097841 (PMC10178039; doi:10.3390/ijms24097841)
Supplement: Supplementary file 1 [file ijms-24-07841-s001.zip › ijms-2345119-supplementary.pdf]

Supplementary Materials for

New pecJ-*n* (*n* = 1, 2) basis sets for selenium atom purposed for the calculations of NMR spin-spin coupling constants involving selenium

by Yuriy Yu. Rusakov and Irina L. Rusakova

Table S1. Equilibrium geometries of two fitting molecules SeH_2 and Se=C and that of testing compounds **1**-**7** (the level of optimization is M06-2X/pc-3).

Molecule	Cartesian Coordinates, Å			
SeH2	Se	0.000000	0.000000	0.056114
	H	0.000000	1.037025	-0.953934
	H	0.000000	-1.037025	-0.953934
Se=C	Se	0.000000	0.000000	0.000000
	C	0.000000	0.000000	1.662334
Me_2Se_2 (**1**)	Se	1.100384	-0.314408	-0.365502
	Se	-1.100384	-0.314408	0.365502
	C	1.833865	1.105028	0.761441
	C	-1.833865	1.105028	-0.761441
	H	-2.886182	1.187824	-0.501994
	H	-1.327234	2.040257	-0.551343
	H	-1.726611	0.831620	-1.804492
	H	2.886182	1.187825	0.501994
	H	1.327233	2.040257	0.551343
	H	1.726612	0.831620	1.804492
MeSe-C≡N (**2**)	Se	0.000000	0.627734	0.000000
	C	-1.768006	-0.210961	0.000000
	C	0.960919	-0.946889	0.000000
	N	1.582618	-1.914747	0.000000
	H	-2.468657	0.618486	0.000000
	H	-1.883574	-0.805551	-0.897914
	H	-1.883574	-0.805551	0.897914
MeSeH (**3**)	Se	-0.031519	-0.420629	0.000000
	C	-0.031519	1.535877	0.000000
	H	-1.075925	1.832110	0.000000
	H	0.454225	1.910104	0.893578

	H 0.454225 1.910104 -0.893578<br>H 1.428243 -0.566192 0.000000
Me2P(Se)Cl (**4**)	Se 0.604223 -1.487019 0.000000<br>C 0.604223 1.472323 -1.445083<br>C 0.604223 1.472323 1.445083<br>P 0.074674 0.528807 0.000000<br>Cl -1.953068 0.887047 0.000000<br>H 0.205180 0.992997 -2.335231<br>H 0.246799 2.497551 -1.375522<br>H 1.691921 1.448872 -1.479959<br>H 0.205180 0.992997 2.335231<br>H 1.691921 1.448872 1.479959<br>H 0.246799 2.497551 1.375522
C4H4Se (**5**)	Se 0.000000 0.000000 0.907629<br>C 0.000000 1.282615 -0.439200<br>C 0.000000 0.714941 -1.669269<br>C 0.000000 -0.714941 -1.669269<br>C 0.000000 -1.282615 -0.439200<br>H 0.000000 2.332883 -0.200899<br>H 0.000000 1.299798 -2.577980<br>H 0.000000 -1.299798 -2.577980<br>H 0.000000 -2.332883 -0.200899
SePMe3 (**6**)	Se 0.000000 0.000000 1.446013<br>C 0.000000 1.657972 -1.402209<br>C -1.435846 -0.828986 -1.402209<br>C 1.435846 -0.828986 -1.402209<br>P 0.000000 0.000000 -0.670630<br>H 0.885133 2.191102 -1.063283<br>H 0.000000 1.592014 -2.489722<br>H -0.885133 2.191102 -1.063283<br>H -1.454984 -1.862098 -1.063283<br>H -2.340116 -0.329004 -1.063283<br>H -1.378725 -0.796007 -2.489722<br>H 1.454984 -1.862098 -1.063283<br>H 1.378725 -0.796007 -2.489722<br>H 2.340116 -0.329004 -1.063283
SiH3SeH (**7**)	Se 0.026466 -0.774339 0.000000<br>Si 0.026466 1.516254 0.000000<br>H 1.450595 1.886490 0.000000<br>H -0.641066 2.037802 -1.204002<br>H -0.641066 2.037802 1.204002<br>H -1.438853 -0.862133 0.000000

Table S2. Basis set pecJ-1 for selenium, CFOUR format.

```
SE:pecJ-1
Property-energy consistent basis set

 4
  0   1   2   3
 11   7   5   3
 16  11   7   3

2.738937E+07  4.117126E+06  5.008352E+05  8.450803E+04  1.940949E+04
5.478217E+03  1.785352E+03  6.442465E+02  2.515808E+02  1.039878E+02
3.331319E+01  1.424322E+01  4.067050E+00  1.754643E+00  3.363182E-01
1.350861E-01

 1.3584E-06  4.2916E-07  0.00000000  0.00000000  0.00000000  0.00000000  0.00000000
 0.00000000  0.00000000  0.00000000  0.00000000
 1.7390E-05  5.4121E-06  0.00000000  0.00000000  0.00000000  0.00000000  0.00000000
 0.00000000  0.00000000  0.00000000  0.00000000
 2.1705E-04  6.8544E-05  0.00000000  0.00000000  0.00000000  0.00000000  0.00000000
 0.00000000  0.00000000  0.00000000  0.00000000
 1.6462E-03  5.1614E-04  0.00000000  0.00000000  0.00000000  0.00000000  0.00000000
 0.00000000  0.00000000  0.00000000  0.00000000
 8.6238E-03  2.7472E-03  0.00000000  0.00000000  0.00000000  0.00000000  0.00000000
 0.00000000  0.00000000  0.00000000  0.00000000
 3.5307E-02  1.1276E-02  0.00000000  0.00000000  0.00000000  0.00000000  0.00000000
 0.00000000  0.00000000  0.00000000  0.00000000
 1.1465E-01  3.9306E-02  0.00000000  0.00000000  0.00000000  0.00000000  0.00000000
 0.00000000  0.00000000  0.00000000  0.00000000
 0.00000000  0.00000000  1.00000000  0.00000000  0.00000000  0.00000000  0.00000000
 0.00000000  0.00000000  0.00000000  0.00000000
 0.00000000  0.00000000  0.00000000  1.00000000  0.00000000  0.00000000  0.00000000
 0.00000000  0.00000000  0.00000000  0.00000000
 0.00000000  0.00000000  0.00000000  0.00000000  1.00000000  0.00000000  0.00000000
 0.00000000  0.00000000  0.00000000  0.00000000
 0.00000000  0.00000000  0.00000000  0.00000000  0.00000000  1.00000000  0.00000000
 0.00000000  0.00000000  0.00000000  0.00000000
```

```
 0.00000000  0.00000000  0.00000000  0.00000000  0.00000000  0.00000000  1.00000000
 0.00000000  0.00000000  0.00000000  0.00000000
 0.00000000  0.00000000  0.00000000  0.00000000  0.00000000  0.00000000  0.00000000
 1.00000000  0.00000000  0.00000000  0.00000000
 0.00000000  0.00000000  0.00000000  0.00000000  0.00000000  0.00000000  0.00000000
 0.00000000  1.00000000  0.00000000  0.00000000
 0.00000000  0.00000000  0.00000000  0.00000000  0.00000000  0.00000000  0.00000000
 0.00000000  0.00000000  1.00000000  0.00000000
 0.00000000  0.00000000  0.00000000  0.00000000  0.00000000  0.00000000  0.00000000
 0.00000000  0.00000000  0.00000000  1.00000000

4.106684E+03  9.693471E+02  3.105258E+02  1.166637E+02  4.812571E+01
2.088559E+01  8.846983E+00  3.839338E+00  1.605191E+00  4.680879E-01
1.490880E-01

 1.4225E-03  5.7021E-04  0.00000000  0.00000000  0.00000000  0.00000000  0.00000000
 1.2057E-02  4.8491E-03  0.00000000  0.00000000  0.00000000  0.00000000  0.00000000
 6.1443E-02  2.4975E-02  0.00000000  0.00000000  0.00000000  0.00000000  0.00000000
 2.0152E-01  8.4407E-02  0.00000000  0.00000000  0.00000000  0.00000000  0.00000000
 4.0415E-01  1.8090E-01  0.00000000  0.00000000  0.00000000  0.00000000  0.00000000
 3.8893E-01  1.7468E-01  0.00000000  0.00000000  0.00000000  0.00000000  0.00000000
 0.00000000  0.00000000  1.00000000  0.00000000  0.00000000  0.00000000  0.00000000
 0.00000000  0.00000000  0.00000000  1.00000000  0.00000000  0.00000000  0.00000000
 0.00000000  0.00000000  0.00000000  0.00000000  1.00000000  0.00000000  0.00000000
 0.00000000  0.00000000  0.00000000  0.00000000  0.00000000  1.00000000  0.00000000
 0.00000000  0.00000000  0.00000000  0.00000000  0.00000000  0.00000000  1.00000000

1.200481E+02  3.552605E+01  1.289256E+01  4.991605E+00  1.889243E+00
6.476664E-01  1.788622E-01

 1.4755E-02  0.00000000  0.00000000  0.00000000  0.00000000
 9.1608E-02  0.00000000  0.00000000  0.00000000  0.00000000
 2.7516E-01  0.00000000  0.00000000  0.00000000  0.00000000
 0.00000000  1.00000000  0.00000000  0.00000000  0.00000000
 0.00000000  0.00000000  1.00000000  0.00000000  0.00000000
 0.00000000  0.00000000  0.00000000  1.00000000  0.00000000
 0.00000000  0.00000000  0.00000000  0.00000000  1.00000000

7.528700E+00  1.282966E+00  3.328661E-01

 1.00000000  0.00000000  0.00000000
 0.00000000  1.00000000  0.00000000
 0.00000000  0.00000000  1.00000000
```

Table S3. Basis set pecJ-2 for selenium, CFOUR format.

```
SE:pecJ-2
Property-energy consistent basis set

 4
  0   1   2   3   4
 15   8   5   4   1
 22  13   8   4   1

4.443822E+08  7.050053E+07  1.041355E+07  1.376983E+06  3.335063E+05
9.200230E+04  2.967395E+04  1.070423E+04  4.148222E+03  1.725619E+03
```

```
7.670764E+02  3.540569E+02  1.675661E+02  8.094489E+01  3.573295E+01
1.748383E+01  8.669421E+00  3.664945E+00  1.624862E+00  5.344704E-01
2.507232E-01  9.266461E-02

 5.6619E-08  1.7694E-08  0.00000000  0.00000000  0.00000000  0.00000000  0.00000000
 0.00000000  0.00000000  0.00000000  0.00000000  0.00000000  0.00000000  0.00000000
 0.00000000
 3.3187E-07  1.0380E-07  0.00000000  0.00000000  0.00000000  0.00000000  0.00000000
 0.00000000  0.00000000  0.00000000  0.00000000  0.00000000  0.00000000  0.00000000
 0.00000000
 5.7928E-06  1.8293E-06  0.00000000  0.00000000  0.00000000  0.00000000  0.00000000
 0.00000000  0.00000000  0.00000000  0.00000000  0.00000000  0.00000000  0.00000000
 0.00000000
 5.0582E-05  1.5827E-05  0.00000000  0.00000000  0.00000000  0.00000000  0.00000000
 0.00000000  0.00000000  0.00000000  0.00000000  0.00000000  0.00000000  0.00000000
 0.00000000
 2.4779E-04  7.8103E-05  0.00000000  0.00000000  0.00000000  0.00000000  0.00000000
 0.00000000  0.00000000  0.00000000  0.00000000  0.00000000  0.00000000  0.00000000
 0.00000000
 1.1207E-03  3.4962E-04  0.00000000  0.00000000  0.00000000  0.00000000  0.00000000
 0.00000000  0.00000000  0.00000000  0.00000000  0.00000000  0.00000000  0.00000000
 0.00000000
 4.0164E-03  1.2647E-03  0.00000000  0.00000000  0.00000000  0.00000000  0.00000000
 0.00000000  0.00000000  0.00000000  0.00000000  0.00000000  0.00000000  0.00000000
 0.00000000
 1.3005E-02  4.1095E-03  0.00000000  0.00000000  0.00000000  0.00000000  0.00000000
 0.00000000  0.00000000  0.00000000  0.00000000  0.00000000  0.00000000  0.00000000
 0.00000000
 3.7719E-02  1.2275E-02  0.00000000  0.00000000  0.00000000  0.00000000  0.00000000
 0.00000000  0.00000000  0.00000000  0.00000000  0.00000000  0.00000000  0.00000000
 0.00000000
 0.00000000  0.00000000  1.00000000  0.00000000  0.00000000  0.00000000  0.00000000
 0.00000000  0.00000000  0.00000000  0.00000000  0.00000000  0.00000000  0.00000000
 0.00000000
 0.00000000  0.00000000  0.00000000  1.00000000  0.00000000  0.00000000  0.00000000
 0.00000000  0.00000000  0.00000000  0.00000000  0.00000000  0.00000000  0.00000000
 0.00000000
 0.00000000  0.00000000  0.00000000  0.00000000  1.00000000  0.00000000  0.00000000
 0.00000000  0.00000000  0.00000000  0.00000000  0.00000000  0.00000000  0.00000000
 0.00000000
 0.00000000  0.00000000  0.00000000  0.00000000  0.00000000  1.00000000  0.00000000
 0.00000000  0.00000000  0.00000000  0.00000000  0.00000000  0.00000000  0.00000000
 0.00000000
 0.00000000  0.00000000  0.00000000  0.00000000  0.00000000  0.00000000  1.00000000
 0.00000000  0.00000000  0.00000000  0.00000000  0.00000000  0.00000000  0.00000000
 0.00000000
 0.00000000  0.00000000  0.00000000  0.00000000  0.00000000  0.00000000  0.00000000
 1.00000000  0.00000000  0.00000000  0.00000000  0.00000000  0.00000000  0.00000000
 0.00000000
 0.00000000  0.00000000  0.00000000  0.00000000  0.00000000  0.00000000  0.00000000
 0.00000000  1.00000000  0.00000000  0.00000000  0.00000000  0.00000000  0.00000000
 0.00000000
 0.00000000  0.00000000  0.00000000  0.00000000  0.00000000  0.00000000  0.00000000
 0.00000000  0.00000000  1.00000000  0.00000000  0.00000000  0.00000000  0.00000000
 0.00000000
 0.00000000  0.00000000  0.00000000  0.00000000  0.00000000  0.00000000  0.00000000
 0.00000000  0.00000000  0.00000000  1.00000000  0.00000000  0.00000000  0.00000000
 0.00000000
 0.00000000  0.00000000  0.00000000  0.00000000  0.00000000  0.00000000  0.00000000
 0.00000000  0.00000000  0.00000000  0.00000000  1.00000000  0.00000000  0.00000000
 0.00000000
 0.00000000  0.00000000  0.00000000  0.00000000  0.00000000  0.00000000  0.00000000
 0.00000000  0.00000000  0.00000000  0.00000000  0.00000000  1.00000000  0.00000000
 0.00000000
 0.00000000  0.00000000  0.00000000  0.00000000  0.00000000  0.00000000  0.00000000
 0.00000000  0.00000000  0.00000000  0.00000000  0.00000000  0.00000000  1.00000000
 0.00000000
```

```
 0.00000000  0.00000000  0.00000000  0.00000000  0.00000000  0.00000000  0.00000000
 0.00000000  0.00000000  0.00000000  0.00000000  0.00000000  0.00000000  0.00000000
 1.00000000

1.020290E+04  2.385085E+03  7.246974E+02  2.599333E+02  1.042775E+02
4.521224E+01  2.064791E+01  9.300946E+00  4.203860E+00  1.835088E+00
6.372571E-01  2.508116E-01  8.507613E-02

 2.8852E-04  1.1040E-04  0.00000000  0.00000000  0.00000000  0.00000000  0.00000000
 0.00000000
 2.7773E-03  1.0953E-03  0.00000000  0.00000000  0.00000000  0.00000000  0.00000000
 0.00000000
 1.7261E-02  6.8573E-03  0.00000000  0.00000000  0.00000000  0.00000000  0.00000000
 0.00000000
 7.4340E-02  3.0010E-02  0.00000000  0.00000000  0.00000000  0.00000000  0.00000000
 0.00000000
 2.1704E-01  9.1417E-02  0.00000000  0.00000000  0.00000000  0.00000000  0.00000000
 0.00000000
 3.9428E-01  1.7705E-01  0.00000000  0.00000000  0.00000000  0.00000000  0.00000000
 0.00000000
 3.6400E-01  1.6148E-01  0.00000000  0.00000000  0.00000000  0.00000000  0.00000000
 0.00000000
 0.00000000  0.00000000  1.00000000  0.00000000  0.00000000  0.00000000  0.00000000
 0.00000000
 0.00000000  0.00000000  0.00000000  1.00000000  0.00000000  0.00000000  0.00000000
 0.00000000
 0.00000000  0.00000000  0.00000000  0.00000000  1.00000000  0.00000000  0.00000000
 0.00000000
 0.00000000  0.00000000  0.00000000  0.00000000  0.00000000  1.00000000  0.00000000
 0.00000000
 0.00000000  0.00000000  0.00000000  0.00000000  0.00000000  0.00000000  1.00000000
 0.00000000
 0.00000000  0.00000000  0.00000000  0.00000000  0.00000000  0.00000000  0.00000000
 1.00000000

2.753441E+02  7.795287E+01  2.796792E+01  1.122825E+01  4.658535E+00
1.894959E+00  7.145110E-01  2.286113E-01

 2.8912E-03  0.00000000  0.00000000  0.00000000  0.00000000
 2.5978E-02  0.00000000  0.00000000  0.00000000  0.00000000
 1.1369E-01  0.00000000  0.00000000  0.00000000  0.00000000
 2.8390E-01  0.00000000  0.00000000  0.00000000  0.00000000
 0.00000000  1.00000000  0.00000000  0.00000000  0.00000000
 0.00000000  0.00000000  1.00000000  0.00000000  0.00000000
 0.00000000  0.00000000  0.00000000  1.00000000  0.00000000
 0.00000000  0.00000000  0.00000000  0.00000000  1.00000000

9.489329E+00  2.271550E+00  5.063583E-01  1.137481E-01

 1.00000000  0.00000000  0.00000000  0.00000000
 0.00000000  1.00000000  0.00000000  0.00000000
 0.00000000  0.00000000  1.00000000  0.00000000
 0.00000000  0.00000000  0.00000000  1.00000000

2.237599E-01

 1.00000000
```

Table S4. Basis set pecJ-1 for selenium, Dalton format.

```
$ Se
a 34
$ s functions
  16   11    0
```

```
  2.738937E+07  1.3584E-06  4.2916E-07  0.00000000  0.00000000  0.00000000  0.00000000  0.00000000  0.00000000  0.00000000  0.00000000
0.00000000
  4.117126E+06  1.7390E-05  5.4121E-06  0.00000000  0.00000000  0.00000000  0.00000000  0.00000000  0.00000000  0.00000000  0.00000000
0.00000000
  5.008352E+05  2.1705E-04  6.8544E-05  0.00000000  0.00000000  0.00000000  0.00000000  0.00000000  0.00000000  0.00000000  0.00000000
0.00000000
  8.450803E+04  1.6462E-03  5.1614E-04  0.00000000  0.00000000  0.00000000  0.00000000  0.00000000  0.00000000  0.00000000  0.00000000
0.00000000
  1.940949E+04  8.6238E-03  2.7472E-03  0.00000000  0.00000000  0.00000000  0.00000000  0.00000000  0.00000000  0.00000000  0.00000000
0.00000000
  5.478217E+03  3.5307E-02  1.1276E-02  0.00000000  0.00000000  0.00000000  0.00000000  0.00000000  0.00000000  0.00000000  0.00000000
0.00000000
  1.785352E+03  1.1465E-01  3.9306E-02  0.00000000  0.00000000  0.00000000  0.00000000  0.00000000  0.00000000  0.00000000  0.00000000
0.00000000
  6.442465E+02  0.00000000  0.00000000  1.00000000  0.00000000  0.00000000  0.00000000  0.00000000  0.00000000  0.00000000  0.00000000
0.00000000
  2.515808E+02  0.00000000  0.00000000  0.00000000  1.00000000  0.00000000  0.00000000  0.00000000  0.00000000  0.00000000  0.00000000
0.00000000
  1.039878E+02  0.00000000  0.00000000  0.00000000  0.00000000  1.00000000  0.00000000  0.00000000  0.00000000  0.00000000  0.00000000
0.00000000
  3.331319E+01  0.00000000  0.00000000  0.00000000  0.00000000  0.00000000  1.00000000  0.00000000  0.00000000  0.00000000  0.00000000
0.00000000
  1.424322E+01  0.00000000  0.00000000  0.00000000  0.00000000  0.00000000  0.00000000  1.00000000  0.00000000  0.00000000  0.00000000
0.00000000
  4.067050E+00  0.00000000  0.00000000  0.00000000  0.00000000  0.00000000  0.00000000  0.00000000  1.00000000  0.00000000  0.00000000
0.00000000
  1.754643E+00  0.00000000  0.00000000  0.00000000  0.00000000  0.00000000  0.00000000  0.00000000  0.00000000  1.00000000  0.00000000
0.00000000
  3.363182E-01  0.00000000  0.00000000  0.00000000  0.00000000  0.00000000  0.00000000  0.00000000  0.00000000  0.00000000  1.00000000
0.00000000
  1.350861E-01  0.00000000  0.00000000  0.00000000  0.00000000  0.00000000  0.00000000  0.00000000  0.00000000  0.00000000  0.00000000
1.00000000
$ p functions
  11   7   0
  4.106684E+03  1.4225E-03  5.7021E-04  0.00000000  0.00000000  0.00000000  0.00000000  0.00000000
  9.693471E+02  1.2057E-02  4.8491E-03  0.00000000  0.00000000  0.00000000  0.00000000  0.00000000
  3.105258E+02  6.1443E-02  2.4975E-02  0.00000000  0.00000000  0.00000000  0.00000000  0.00000000
  1.166637E+02  2.0152E-01  8.4407E-02  0.00000000  0.00000000  0.00000000  0.00000000  0.00000000
  4.812571E+01  4.0415E-01  1.8090E-01  0.00000000  0.00000000  0.00000000  0.00000000  0.00000000
  2.088559E+01  3.8893E-01  1.7468E-01  0.00000000  0.00000000  0.00000000  0.00000000  0.00000000
  8.846983E+00  0.00000000  0.00000000  1.00000000  0.00000000  0.00000000  0.00000000  0.00000000
  3.839338E+00  0.00000000  0.00000000  0.00000000  1.00000000  0.00000000  0.00000000  0.00000000
  1.605191E+00  0.00000000  0.00000000  0.00000000  0.00000000  1.00000000  0.00000000  0.00000000
  4.680879E-01  0.00000000  0.00000000  0.00000000  0.00000000  0.00000000  1.00000000  0.00000000
  1.490880E-01  0.00000000  0.00000000  0.00000000  0.00000000  0.00000000  0.00000000  1.00000000
$ d functions
  7   5   0
  1.200481E+02  1.4755E-02  0.00000000  0.00000000  0.00000000  0.00000000
  3.552605E+01  9.1608E-02  0.00000000  0.00000000  0.00000000  0.00000000
  1.289256E+01  2.7516E-01  0.00000000  0.00000000  0.00000000  0.00000000
  4.991605E+00  0.00000000  1.00000000  0.00000000  0.00000000  0.00000000
  1.889243E+00  0.00000000  0.00000000  1.00000000  0.00000000  0.00000000
  6.476664E-01  0.00000000  0.00000000  0.00000000  1.00000000  0.00000000
  1.788622E-01  0.00000000  0.00000000  0.00000000  0.00000000  1.00000000
$ f functions
  3   3   0
  7.528700E+00  1.00000000  0.00000000  0.00000000
  1.282966E+00  0.00000000  1.00000000  0.00000000
  3.328661E-01  0.00000000  0.00000000  1.00000000
```

Table S5. Basis set pecJ-2 for selenium, Dalton format.

```
$ Se
a 34
$ s functions
  22   15   0
  4.443822E+08  5.6619E-08  1.7694E-08  0.00000000  0.00000000  0.00000000  0.00000000
  0.00000000     0.00000000  0.00000000  0.00000000  0.00000000  0.00000000  0.00000000
  0.00000000     0.00000000
  7.050053E+07  3.3187E-07  1.0380E-07  0.00000000  0.00000000  0.00000000  0.00000000
  0.00000000     0.00000000  0.00000000  0.00000000  0.00000000  0.00000000  0.00000000
  0.00000000     0.00000000
```

```
  1.041355E+07  5.7928E-06  1.8293E-06  0.00000000  0.00000000  0.00000000  0.00000000
  0.00000000    0.00000000  0.00000000  0.00000000  0.00000000  0.00000000  0.00000000
  0.00000000    0.00000000
  1.376983E+06  5.0582E-05  1.5827E-05  0.00000000  0.00000000  0.00000000  0.00000000
  0.00000000    0.00000000  0.00000000  0.00000000  0.00000000  0.00000000  0.00000000
  0.00000000    0.00000000
  3.335063E+05  2.4779E-04  7.8103E-05  0.00000000  0.00000000  0.00000000  0.00000000
  0.00000000    0.00000000  0.00000000  0.00000000  0.00000000  0.00000000  0.00000000
  0.00000000    0.00000000
  9.200230E+04  1.1207E-03  3.4962E-04  0.00000000  0.00000000  0.00000000  0.00000000
  0.00000000    0.00000000  0.00000000  0.00000000  0.00000000  0.00000000  0.00000000
  0.00000000    0.00000000
  2.967395E+04  4.0164E-03  1.2647E-03  0.00000000  0.00000000  0.00000000  0.00000000
  0.00000000    0.00000000  0.00000000  0.00000000  0.00000000  0.00000000  0.00000000
  0.00000000    0.00000000
  1.070423E+04  1.3005E-02  4.1095E-03  0.00000000  0.00000000  0.00000000  0.00000000
  0.00000000    0.00000000  0.00000000  0.00000000  0.00000000  0.00000000  0.00000000
  0.00000000    0.00000000
  4.148222E+03  3.7719E-02  1.2275E-02  0.00000000  0.00000000  0.00000000  0.00000000
  0.00000000    0.00000000  0.00000000  0.00000000  0.00000000  0.00000000  0.00000000
  0.00000000    0.00000000
  1.725619E+03  0.00000000  0.00000000  1.00000000  0.00000000  0.00000000  0.00000000
  0.00000000    0.00000000  0.00000000  0.00000000  0.00000000  0.00000000  0.00000000
  0.00000000    0.00000000
  7.670764E+02  0.00000000  0.00000000  0.00000000  1.00000000  0.00000000  0.00000000
  0.00000000    0.00000000  0.00000000  0.00000000  0.00000000  0.00000000  0.00000000
  0.00000000    0.00000000
  3.540569E+02  0.00000000  0.00000000  0.00000000  0.00000000  1.00000000  0.00000000
  0.00000000    0.00000000  0.00000000  0.00000000  0.00000000  0.00000000  0.00000000
  0.00000000    0.00000000
  1.675661E+02  0.00000000  0.00000000  0.00000000  0.00000000  0.00000000  1.00000000
  0.00000000    0.00000000  0.00000000  0.00000000  0.00000000  0.00000000  0.00000000
  0.00000000    0.00000000
  8.094489E+01  0.00000000  0.00000000  0.00000000  0.00000000  0.00000000  0.00000000
  1.00000000    0.00000000  0.00000000  0.00000000  0.00000000  0.00000000  0.00000000
  0.00000000    0.00000000
  3.573295E+01  0.00000000  0.00000000  0.00000000  0.00000000  0.00000000  0.00000000
  0.00000000    1.00000000  0.00000000  0.00000000  0.00000000  0.00000000  0.00000000
  0.00000000    0.00000000
  1.748383E+01  0.00000000  0.00000000  0.00000000  0.00000000  0.00000000  0.00000000
  0.00000000    0.00000000  1.00000000  0.00000000  0.00000000  0.00000000  0.00000000
  0.00000000    0.00000000
  8.669421E+00  0.00000000  0.00000000  0.00000000  0.00000000  0.00000000  0.00000000
  0.00000000    0.00000000  0.00000000  1.00000000  0.00000000  0.00000000  0.00000000
  0.00000000    0.00000000
  3.664945E+00  0.00000000  0.00000000  0.00000000  0.00000000  0.00000000  0.00000000
  0.00000000    0.00000000  0.00000000  0.00000000  1.00000000  0.00000000  0.00000000
  0.00000000    0.00000000
  1.624862E+00  0.00000000  0.00000000  0.00000000  0.00000000  0.00000000  0.00000000
  0.00000000    0.00000000  0.00000000  0.00000000  0.00000000  1.00000000  0.00000000
  0.00000000    0.00000000
  5.344704E-01  0.00000000  0.00000000  0.00000000  0.00000000  0.00000000  0.00000000
  0.00000000    0.00000000  0.00000000  0.00000000  0.00000000  0.00000000  1.00000000
  0.00000000    0.00000000
  2.507232E-01  0.00000000  0.00000000  0.00000000  0.00000000  0.00000000  0.00000000
  0.00000000    0.00000000  0.00000000  0.00000000  0.00000000  0.00000000  0.00000000
  1.00000000    0.00000000
  9.266461E-02  0.00000000  0.00000000  0.00000000  0.00000000  0.00000000  0.00000000
  0.00000000    0.00000000  0.00000000  0.00000000  0.00000000  0.00000000  0.00000000
  0.00000000    1.00000000
$ p functions
  13   8   0
  1.020290E+04  2.8852E-04  1.1040E-04  0.00000000  0.00000000  0.00000000  0.00000000
  0.00000000    0.00000000
  2.385085E+03  2.7773E-03  1.0953E-03  0.00000000  0.00000000  0.00000000  0.00000000
  0.00000000    0.00000000
  7.246974E+02  1.7261E-02  6.8573E-03  0.00000000  0.00000000  0.00000000  0.00000000
  0.00000000    0.00000000
  2.599333E+02  7.4340E-02  3.0010E-02  0.00000000  0.00000000  0.00000000  0.00000000
  0.00000000    0.00000000
```

```
  1.042775E+02  2.1704E-01  9.1417E-02  0.00000000  0.00000000  0.00000000  0.00000000
  0.00000000     0.00000000
  4.521224E+01  3.9428E-01  1.7705E-01  0.00000000  0.00000000  0.00000000  0.00000000
  0.00000000     0.00000000
  2.064791E+01  3.6400E-01  1.6148E-01  0.00000000  0.00000000  0.00000000  0.00000000
  0.00000000     0.00000000
  9.300946E+00  0.00000000  0.00000000  1.00000000  0.00000000  0.00000000  0.00000000
  0.00000000     0.00000000
  4.203860E+00  0.00000000  0.00000000  0.00000000  1.00000000  0.00000000  0.00000000
  0.00000000     0.00000000
  1.835088E+00  0.00000000  0.00000000  0.00000000  0.00000000  1.00000000  0.00000000
  0.00000000     0.00000000
  6.372571E-01  0.00000000  0.00000000  0.00000000  0.00000000  0.00000000  1.00000000
  0.00000000     0.00000000
  2.508116E-01  0.00000000  0.00000000  0.00000000  0.00000000  0.00000000  0.00000000
  1.00000000     0.00000000
  8.507613E-02  0.00000000  0.00000000  0.00000000  0.00000000  0.00000000  0.00000000
  0.00000000     1.00000000
$ d functions
   8   5   0
  2.753441E+02  2.8912E-03  0.00000000  0.00000000  0.00000000  0.00000000
  7.795287E+01  2.5978E-02  0.00000000  0.00000000  0.00000000  0.00000000
  2.796792E+01  1.1369E-01  0.00000000  0.00000000  0.00000000  0.00000000
  1.122825E+01  2.8390E-01  0.00000000  0.00000000  0.00000000  0.00000000
  4.658535E+00  0.00000000  1.00000000  0.00000000  0.00000000  0.00000000
  1.894959E+00  0.00000000  0.00000000  1.00000000  0.00000000  0.00000000
  7.145110E-01  0.00000000  0.00000000  0.00000000  1.00000000  0.00000000
  2.286113E-01  0.00000000  0.00000000  0.00000000  0.00000000  1.00000000
$ f functions
   4   4   0
  9.489329E+00  1.00000000  0.00000000  0.00000000  0.00000000
  2.271550E+00  0.00000000  1.00000000  0.00000000  0.00000000
  5.063583E-01  0.00000000  0.00000000  1.00000000  0.00000000
  1.137481E-01  0.00000000  0.00000000  0.00000000  1.00000000
$ g functions
   1   1   0
  2.237599E-01  1.00000000
```

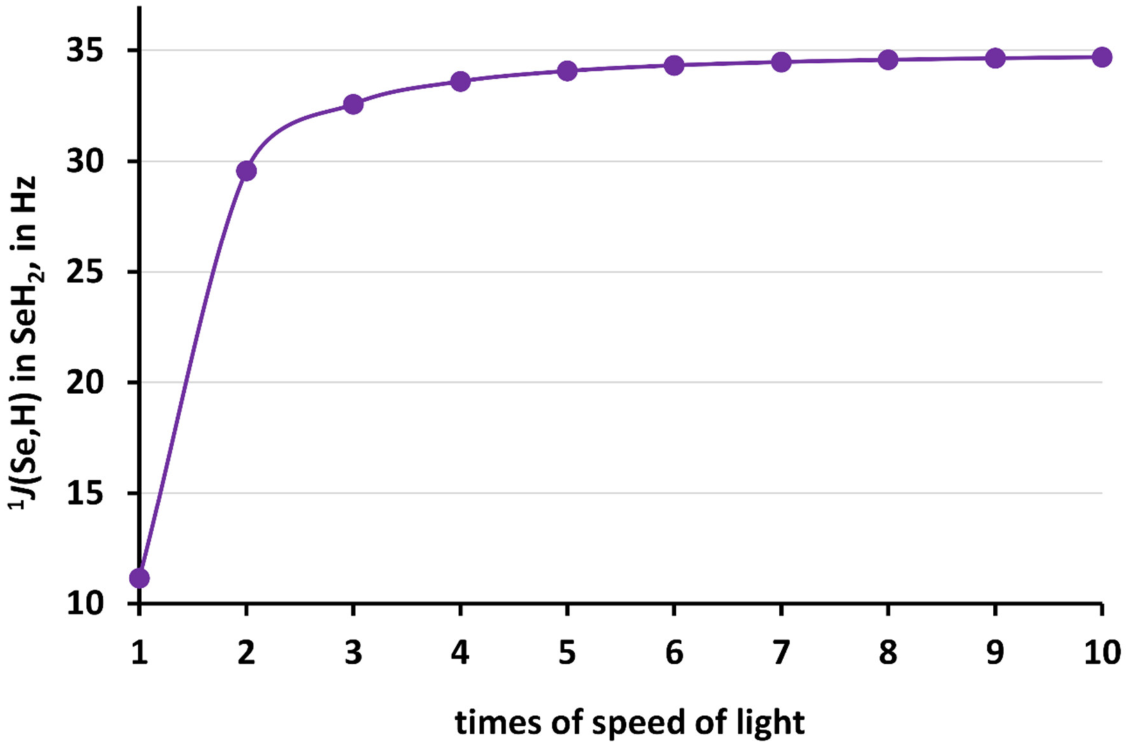

Figure S1. The convergence of ^1J(Se,H) in SeH_2 calculated at the 4-component DFT(SVWN5)//pecJ-2(uc) level with increasing the speed of light by 1 (totally relativistic calculation) to 10 ("10c limit scheme") times.

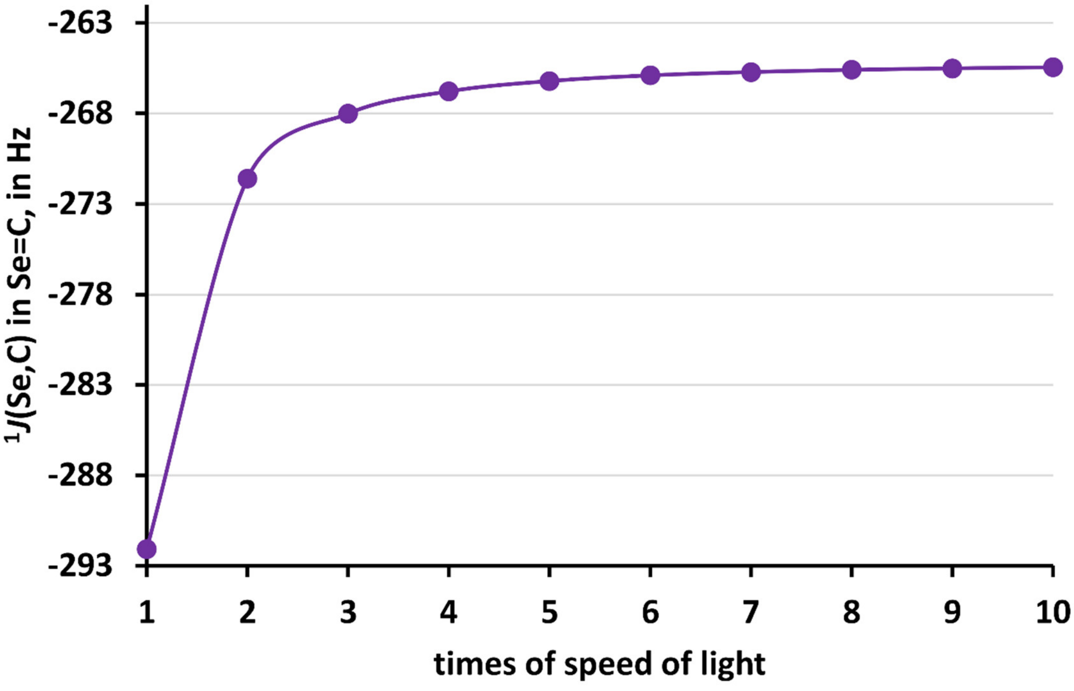

Figure S2. The convergence of ^1J(Se,C) in Se=C calculated at the 4-component DFT(SVWN5)//pecJ-2(uc) level with increasing the speed of light by 1 (totally relativistic calculation) to 10 ("10c limit scheme") times.
